# Probiotic candidates for controlling *Paenibacillus larvae*, a causative agent of American foulbrood disease in honey bee

**DOI:** 10.1186/s12866-023-02902-0

**Published:** 2023-05-24

**Authors:** A-Tai Truong, Jeong Eun Kang, Mi-Sun Yoo, Thi Thu Nguyen, So-Youn Youn, Soon-Seek Yoon, Yun Sang Cho

**Affiliations:** 1 Parasitic and InParasitic and Honey Bee Disease Laboratory, Bacterial Disease Division, Department of Animal and Plant Health Research, Animal and Plant Quarantine Agency, Gimcheon, 39660 Republic of Korea; 2 Faculty of Biotechnology, Thai Nguyen University of Sciences, Thai Nguyen, 250000 Vietnam

**Keywords:** *Paenibacillus larvae*, American foulbrood, Probiotics, Gut microbiome, *Apis mellifera*, *Lactobacillus*

## Abstract

**Background:**

American foulbrood (AFB) disease caused by *Paenibacillus larvae* is dangerous, and threatens beekeeping. The eco-friendly treatment method using probiotics is expected to be the prospective method for controlling this pathogen in honey bees. Therefore, this study investigated the bacterial species that have antimicrobial activity against *P. larvae*.

**Results:**

Overall, 67 strains of the gut microbiome were isolated and identified in three phyla; the isolates had the following prevalence rates: Firmicutes 41/67 (61.19%), Actinobacteria 24/67 (35.82%), and Proteobacteria 2/67 (2.99%). Antimicrobial properties against *P. larvae* on agar plates were seen in 20 isolates of the genus *Lactobacillus*, Firmicutes phylum. Six representative strains from each species (*L. apis* HSY8_B25, *L. panisapium* PKH2_L3, *L. melliventris* HSY3_B5, *L. kimbladii* AHS3_B36, *L. kullabergensis* OMG2_B25, and *L. mellis* OMG2_B33) with the largest inhibition zones on agar plates were selected for in vitro larvae rearing challenges. The results showed that three isolates (*L. apis* HSY8_B25, *L. panisapium* PKH2_L3, and *L. melliventris* HSY3_B5) had the potential to be probiotic candidates with the properties of safety to larvae, inhibition against *P. larvae* in infected larvae, and high adhesion ability.

**Conclusions:**

Overall, 20 strains of the genus *Lactobacillus* with antimicrobial properties against *P. larvae* were identified in this study. Three representative strains from different species (*L. apis* HSY8_B25, *L. panisapium* PKH2_L3, and *L. melliventris* HSY3_B5) were evaluated to be potential probiotic candidates and were selected for probiotic development for the prevention of AFB. Importantly, the species *L. panisapium* isolated from larvae was identified with antimicrobial activity for the first time in this study.

**Supplementary Information:**

The online version contains supplementary material available at 10.1186/s12866-023-02902-0.

## Background

Honey bees provide important products to humans, such as honey, pollen, royal jelly, and propolis, and play a vital role in pollinating wild plants and crops [1, 2]. It is estimated that 87.5% of all flowering plants and 50% of global crops are pollinated by honey bees (*Apis mellifera*) [3–5]. However, honey bees are facing great challenges of colony losses of up to 30% in some countries [6, 7]. Pests and diseases are considered the major factors that result in honey bee loss [6]. Therefore, investigating efficient methods for disease control to mitigate colony losses is one of the major concerns in beekeeping.

American foulbrood (AFB) is one of the most destructive diseases of honey bees caused by *Paenibacillus larvae*, a spore-forming, gram-positive bacterium [8]. The spores germinate in the gut of infected larvae 12 h after ingestion and rapidly proliferate to kill the larvae [9]. Spores from dead larvae are spread to the hive by worker bees during the removal of the dead larvae, resulting in the collapse of the infected colony [10]. The traditional method of controlling *P. larvae* is using antibiotics such as oxytetracycline and tylosin tartrate [10]. However, there are great concerns about the use of antibiotics because of the adverse effects on honey bees by disturbing the gut microbiome [11, 12], and the *P. larvae* develop resistance to the antibiotics [13–15]. Therefore, it is necessary to develop an alternative eco-friendly method to control and treat AFB.

The digestive tract of honey bees contains a complex of microbial communities [16, 17]. The microbial composition in the gut varies depending on the food sources, season, geographical region, living stage, and different strains of honey bees [18–21]. The bacterial brood diseases caused by *Melissococcus plutonius* and *P. larvae* could result in changes in the gut bacterial community of honey bees, affecting honey bee health [22, 23]. The gut microbiome, specifically the lactic acid bacteria (LAB), was demonstrated to have a beneficial function on honey bees’ health [24]. In addition, the antimicrobial activity of LAB for biological control of *Melissococcus plutonius* and *P. larvae*, the causative agents of European and American foulbrood, was shown [25, 26]. The LAB belonging to the genus *Lactobacillus* and *Bifidobacterium* were isolated from different sources including the gut of adult bees, brood, brood comb, and honey, and were demonstrated to be the potential probiotic candidates for the inhibition of *P. larvae* [27–30]. Further investigations are necessary to identify bacterial species that inhibit *P. larvae*, are safe to honey bee larvae, and meet the requirements for probiotic bacteria.

Accordingly, this study was conducted to isolate the gut bacteria from honey bees *Apis mellifera* and investigate the potential bacterial strains for *P. larvae* inhibition. The evaluation of potential probiotic candidates for AFB disease treatment was performed using the in vitro rearing larvae method.

## Results

### Isolation of gut microbiome

**Fig. 1:**
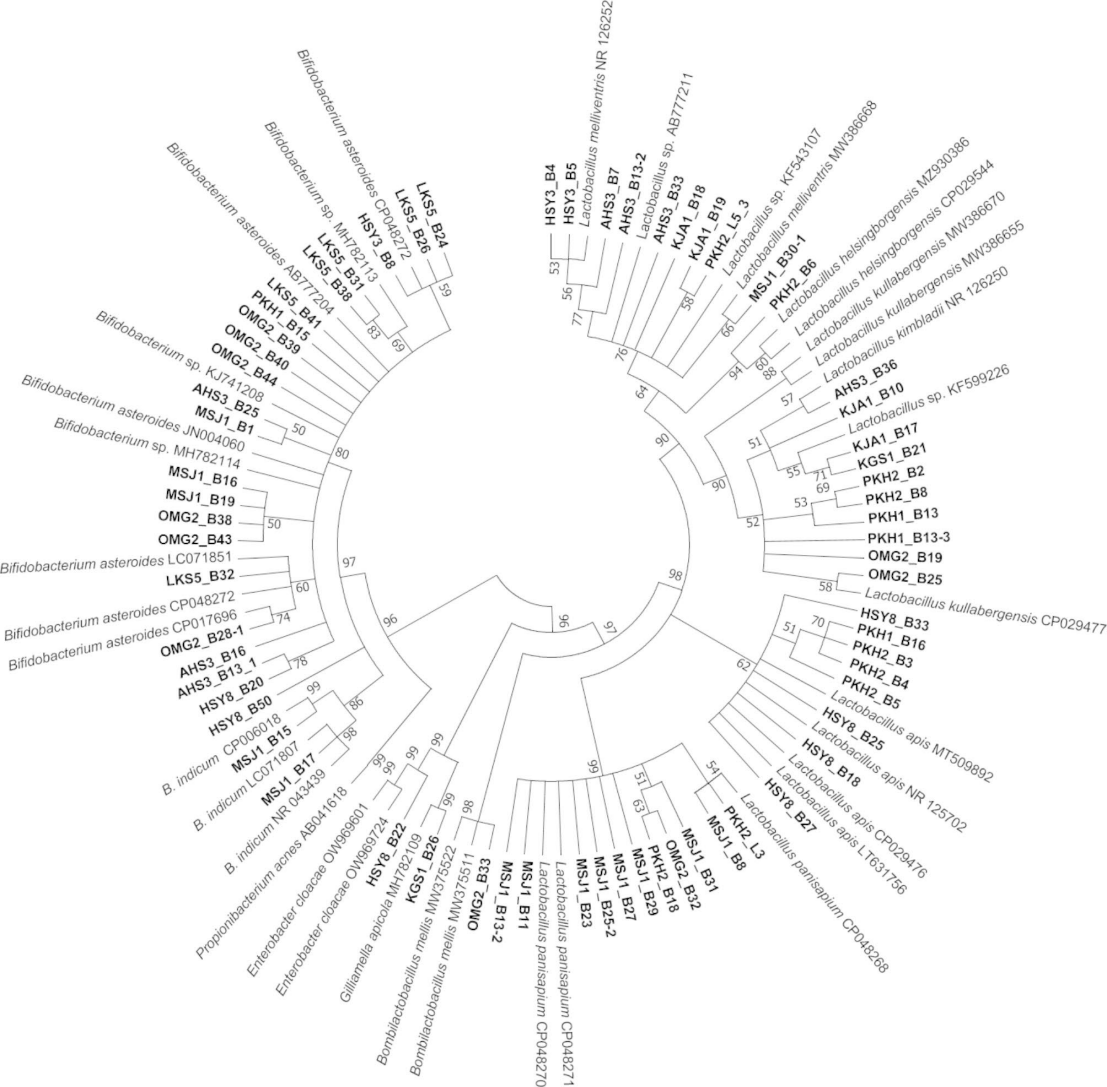
**Phylogenetic tree of isolated bacterial strains.** A maximum likelihood tree was created using the 16 S rRNA gene of 67 isolates with bootstrapping 500. The names of the isolated strains are written in bold, and the reference species name with NCBI accession number is given. Only the percentage of bootstrap ≥ 50% is shown

Overall, 67 strains of honey bee gut bacteria were isolated and identified to belong to three phyla, with the prevalence of the isolates in descending order as follows: Firmicutes 41/67 (61.19%), Actinobacteria 24/67 (35.82%), and Proteobacteria 2/67 (2.99%). The Firmicutes phylum consisted of only one genus, *Lactobacillus*, with six identified species, including *L. panisapium* (9 isolates), *L. apis* (4), *L. mellis* (3), *L. melliventris* (3), *L. kimbladii* (1), and *L. kullabergensis* (1), and other 20 unidentified strains (Fig. 1; Supplementary Table S1). Actinobacteria phylum had one genus, *Bifidobacterium*, with two species (*B. asteroids* and *B. indicum*) and 17 unclassified strains. Meanwhile, the Proteobacteria phylum contained two genera, *Gilliamella* (*G. apicola*) and *Enterobacter* (Fig. 1; Supplementary Table S1).

### Antimicrobial activity of isolated lactic acid bacteria

**Fig. 2:**
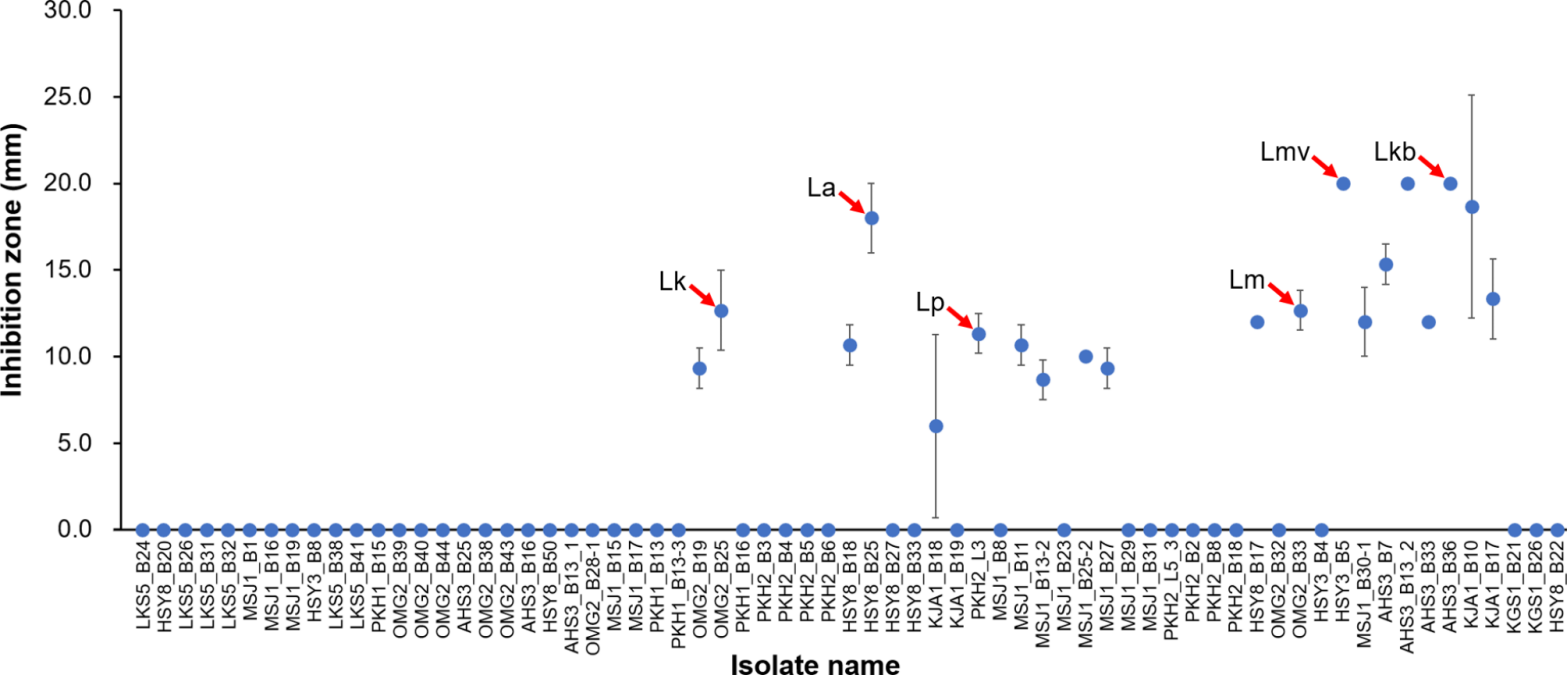
**Antimicrobial activity of isolated bacteria against** ***P. larvae*** **identified by inhibition zone on an agar plate** Twenty of the 67 isolates showed antimicrobial activity with different sizes of inhibition zone, and six of the 20 isolates (indicated by arrows) belonging to different species were selected as probiotic candidates; the selected strains were OMG2_B25 (*L. kullabergensis; Lk*), HSY8_B25 (*L. apis; La*), PKH2_L3 (*L. panisapium; Lp*), OMG2_B33 (*L. mellis; Lm*), HSY3_B5 (*L. melliventris; Lmv*), and AHS3_B36 (*L. kimbladii; Lkb*)

The antimicrobial effect of the isolated strains was evaluated based on the inhibition zone observed surrounding the isolated strains against *P. larvae*, and 20 of 67 isolates of only one genus, *Lactobacillus*, showed antimicrobial activity (Fig. 2; Supplementary Figure S2; Supplementary Table S1). Six representative strains of the six identified species with the largest inhibition zone were selected to evaluate probiotic candidates (Fig. 2). The six strains consisted of OMG2_B25 (*L. kullabergensis*), HSY8_B25 (*L. apis*), PKH2_L3 (*L. panisapium*), OMG2_B33 (*L. mellis*), HSY3_B5 (*L. melliventris*), and AHS3_B36 (*L. kimbladii*). Two strains, HSY3_B5 (*L. melliventris*) and AHS3_B36 (*L. kimbladii)*, had the largest size inhibition zone (20.0 ± 0.0 mm), followed by HSY8_B25 (*L. apis*; 18.0 ± 2.0 mm), OMG2_B33 (*L. mellis*; 12.7 ± 1.2 mm), OMG2_B25 (*L. kullabergensis*; 12.7 ± 2.3 mm), and PKH2_L3 (*L. panisapium*; 11.3 ± 1.2 mm) (Fig. 2). Other two strains of *Lactobacillus*, KJA1_B10 and AHS3_B13-2, also showed a large inhibition zone with 18.7 and 20 mm, respectively (Fig. 2; Supplementary Table S1). However, the species names of the two strains were not identified (Fig. 1). Therefore, these two unidentified strains were not selected for further evaluation. Of the six selected probiotic candidates, only one strain, PKH2_L3 (*L. panisapium*), was isolated from larvae, and the other five strains were from adult honey bees (Supplementary Table S1).

### Safety examination of isolated LAB on honey bee larvae

**Fig. 3:**
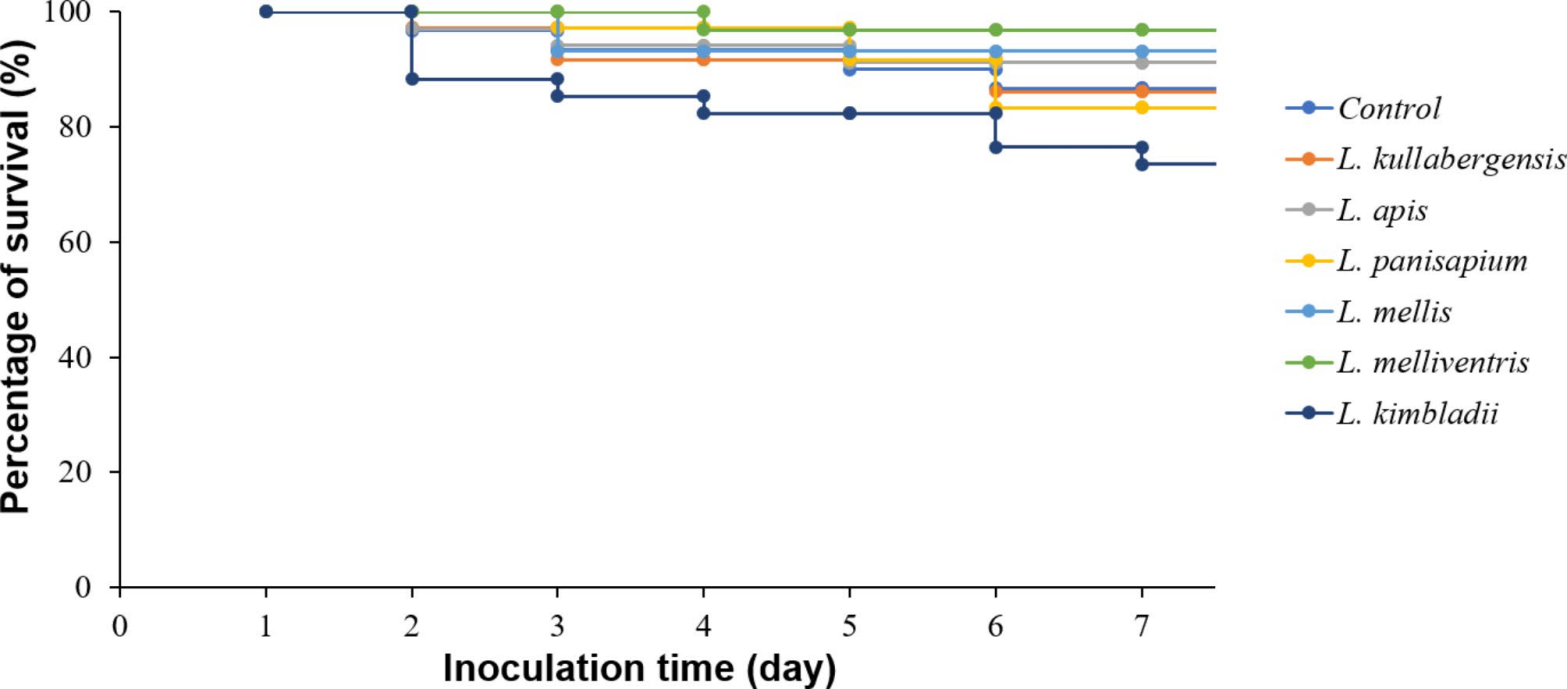
**The survival rate of honey bee larvae that received six isolated LAB strains.** Six species of isolated LAB were supplied to six groups of larvae, the living number of larvae was examined, and the survival rate was calculated daily till day 7. The control group was fed with only a feeding solution

The safety challenge of the six selected LAB to honey bee larvae showed that the survival rates of larval groups that received different strains were not significantly different (*p* = 0.285) during the evaluation period. The larvae received three strains, including *L. apis* HSY8_B25, *L. mellis* OMG2_B33, and *L. melliventris* HSY3_B5, which showed higher survival rates, of 91%, 93%, and 97%, respectively than that of the control group without LAB administration (87%) (Fig. 3). The survival rates of two groups that received *L. kullabergensis* OMG2_B25 and *L. panisapium* PKH2_L3 (86% and 83%, respectively) were comparable with that of the control group. Meanwhile, *L. kimbladii* AHS3_B36 reduced the survival rate of larvae to 74% compared with 87% in the control group. The results demonstrated that the administration of five strains (*L. apis* HSY8_B25, *L. mellis* OMG2_B33, *L. melliventris* HSY3_B5, *L. kullabergensis* OMG2_B25, and *L. panisapium* PKH2_L3) was safe for the honey bee larvae, with the exception of *L. kimbladii* AHS3_B36.

### Biological control of probiotic candidates against ***P. larvae*** in infected larvae

**Fig. 4:**
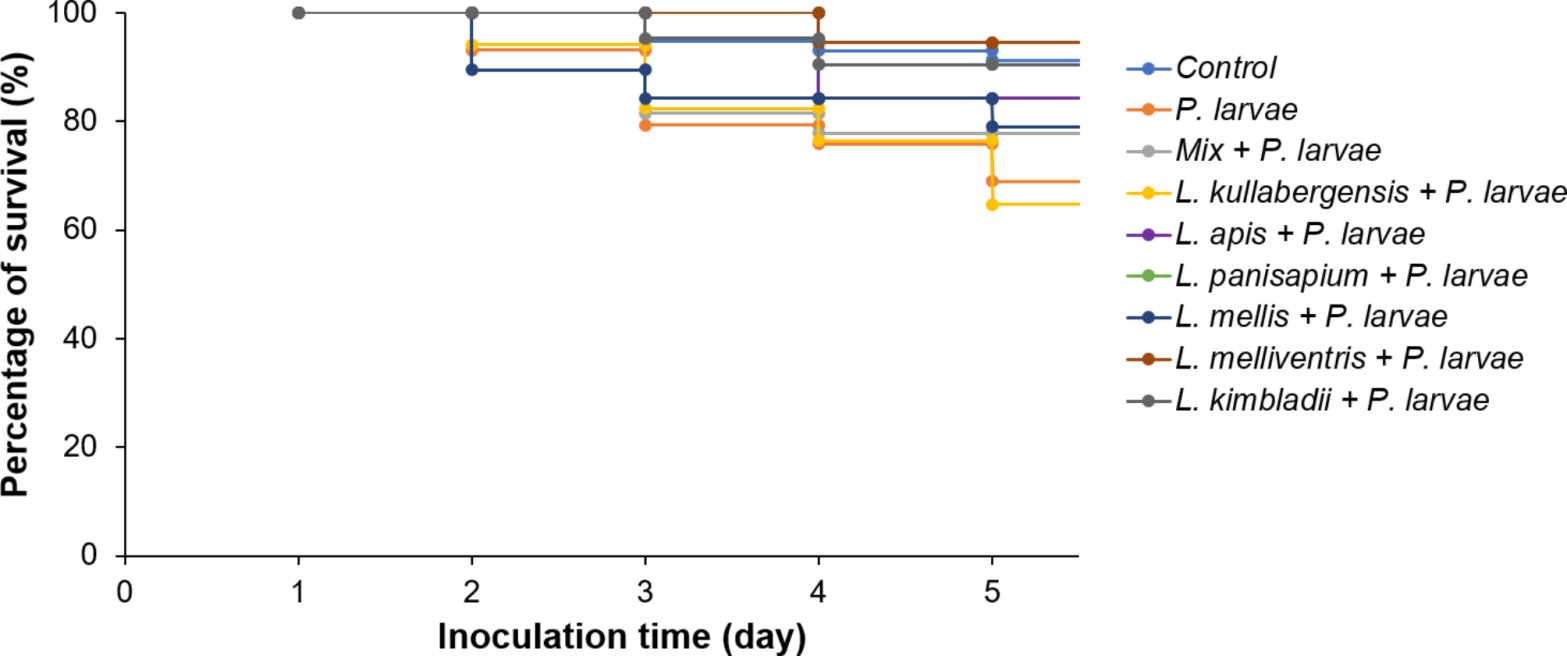
**The survival rate of** ***P. larvae* infected larvae fed with isolated LAB.** The six isolated strains were singly supplied to each group of larvae, and one group was provided with a mixture of six strains. On the first day, after the administration of LAB, the larvae were infected with *P. larvae*. The number of living larvae was examined daily in each group to calculate the survival rate until the fifth day. The control group was neither infected with *P. larvae* nor fed with the LAB. Another group was infected with *P. larvae* without the administration of LAB.

The inhibitory effect of the LAB against *P. larvae* in the infected larvae was evaluated for 5 days after infection. The results showed that the survival rate of larvae fed with *L. kullabergensis* OMG2_B25 (64.71%) was lower than that of the only *P. larvae* infected group (68.97%). Meanwhile, the other five strains and the mixture of the six strains protected larvae by increasing the survival rate. The survival rate after 5 days of infection was 77.78%, 78.95%, 84.21%, 90.48%, 90.48%, and 94.44% for the mixture, *L. mellis* OMG2_B33, *L. apis* HSY8_B25, *L. panisapium* PKH2_L3, *L. kimbladii* AHS3_B36, and *L. melliventris* HSY3_B5, respectively (Fig. 4). Three species, *L. panisapium* PKH2_L3, *L. kimbladii* AHS3_B36, and *L. melliventris* HSY3_B5, helped increase the survival rate to the same level as that of the control group without *P. larvae* infection and no LAB administration (91.23%; *p* = 0.865).

DNA copy number of *P. larvae* in different groups was calculated based on the Ct value of *P. larvae* detection in real-time polymerase chain reaction (PCR) (Table 1). The results demonstrated that the mixture of six LAB strains had the highest inhibition to *P. larvae* with the lowest number of *P. larvae* DNA copies (8.37 × 10^5^ copies), followed by the group fed with *L. panisapium* PKH2_L3 (1.42 × 10^6^ copies), *L. apis* HSY8_B25 (3.64 × 10^6^ copies), *L. kullabergensis* OMG2_B25 (4.56 × 10^6^ copies), *L. mellis* OMG2_B33 (5.49 × 10^6^ copies), *L. melliventris* HSY3_B5 (6.93 × 10^6^ copies), and *L. kimbladii* AHS3_B36 (1.07 × 10^6^ copies). Meanwhile, the *P. larvae* infected group without LAB feeding showed the highest number of *P. larvae* DNA copies, 1.32 × 10^7^ copies. The control group, with no *P. larvae* infection and no LAB administration, showed negative results in *P. larvae* detection (Table 1).

**Table 1:** Quantification of *P. larvae* DNA in larval groups that received different strains of LAB

Group	Only *P. larvae*	Mixture of LAB + *P. larvae*	*L. kullabergensis + P. larvae*	*L. apis + P. larvae*	*L. panisapium + P. larvae*	*L. mellis + P. larvae*	*L. melliventris + P. larvae*	*L. kimbladii + P. larvae*	No *P. larvae*, no LAB
Ct value	18.46	22.49	20.01	20.34	21.72	19.74	19.4	18.74	Negative
*P. larvae* DNA copy	1.32 × 10^7^	8.37 × 10^5^	4.56 × 10^6^	3.64 × 10^6^	1.42 × 10^6^	5.49 × 10^6^	6.93 × 10^6^	1.09 × 10^7^	0

### Hydrophobicity and auto-aggregation

The Bacterial Adherence to Hydrocarbons (BATH) assay results showed that the adhesion ability of the six LAB strains was different. *L. kullabergensis* OMG2_B25 and *L. mellis* OMG2_B33 demonstrated low hydrophobicity with adhesion to toluene (22.10% and 14.83%) and xylene (19.27% and 9.16%), respectively. Meanwhile, the other four strains showed moderate to high hydrophobicity, with the adhesion to toluene ranging from 56.16% (*L. melliventris* HSY3_B5) to 71.03% (*L. kimbladii* AHS3_B36) and the adhesion to xylene ranging from 50.55% (*L. melliventris* HSY3_B5) to 70.09% (*L. panisapium* PKH2_L3; Table 2).

**Table 2:** Adhesion to toluene and xylene of the six isolated lactic acid bacteria

Hydrophobicity (%) Toluene						
Time (min)	*L. kullabergensis* (OMG2_B25*)*	*L. apis* (HSY8_B25)	*L. panisapium* (PKH2_L3)	*L. mellis* (OMG2_B33)	*L. melliventris* (HSY3_B5)	*L. kimbladii* (AHS3_B36)
60	22.10 ± 1.48	68.70 ± 0.39	58.19 ± 1.20	14.83 ± 2.09	56.16 ± 0.84	71.03 ± 0.85
Hydrophobicity (%) Xylene						
60	19.27 ± 2.00	55.45 ± 1.10	70.09 ± 0.45	9.16 ± 1.77	50.55 ± 1.70	56.70 ± 1.15

The auto-aggregation results showed that there was no significant difference (*p* = 0.978) in auto-aggregation percentage (AA%) among the six isolated LAB. All strains showed progressive aggregation over time. The AA% after 24 h was all above 50%, with the lowest value of 82.80% (*L. panisapium* PKH2_L3) and the highest value of 90.21% (*L. melliventris* HSY3_B5) (Table 3). The *L. melliventris* HSY3_B5 showed the fastest auto-aggregation ability with 22.90% at 1 h and reached 87.49% after 5 h, followed by *L. mellis* OMG2_B33 (69.46%), and *L. kimbladii* AHS3_B36 (53.02%) (Table 3). However, the other three strains showed a lower auto-aggregation ability with AA% at 5 h of 45.79%, 43.42%, and 40.50% for *L. apis* HSY8_B25, *L. kullabergensis* OMG2_B25, and *L. panisapium* PKH2_L3, respectively (Table 3).

**Table 3:** Auto-aggregation test of the six isolated lactic acid bacteria

Auto-Aggregation (%)						
Time (h)	*L. kullabergensis* (OMG2_B25*)*	*L. apis* (HSY8_B25)	*L. panisapium* (PKH2_L3)	*L. mellis* (OMG2_B33)	*L. melliventris* (HSY3_B5)	*L. kimbladii* (AHS3_B36)
1	9.86 ± 0.31	2.77 ± 1.38	14.45 ± 2.04	18.65 ± 1.08	22.90 ± 1.22	7.61 ± 0.23
5	45.79 ± 1.13	43.42 ± 0.29	40.50 ± 1.45	69.46 ± 1.08	87.49 ± 0.19	53.02 ± 0.20
18	76.45 ± 1.05	77.00 ± 0.81	63.06 ± 0.72	82.06 ± 0.91	89.97 ± 1.56	78.30 ± 0.98
24	86.18 ± 0.29	85.63 ± 0.21	82.80 ± 0.57	89.08 ± 0.47	90.21 ± 0.84	85.03 ± 0.65

## Discussion

The gut microbiome of honey bees was isolated and identified as belonging to the genera *Bifidobacterium*, *Lactobacillus*, *Gilliamella*, and *Enterobacter*, with *Lactobacillus* spp. being the dominant species. These are the major gut microbiome of honey bees [16, 21], of which *Bifidobacterium* and *Lactobacillus* are known to be the lactic acid producers [31] and *Gilliamella* is identified to have a vital role in improving the dietary tolerances of honey bees [32]. The structure of the honey bee gut microbiome varies depending on the geographical region, specific environmental landscape, humidity, temperature, and seasonality [33–35]. The gut bacterial community can be perturbed by pathogen infections as well as miticide and pesticide exposure [36–39]. Dysbiosis of the gut microbiota could lead to an increased susceptibility of honeybees to pathogens [40, 41]. Therefore, it is crucial to understand the microbial structure of the honey bee gut microbiome and select probiotic candidates that can help maintain a healthy balance of the gut microbiota to enhance honey bee immunity.

Honey bee gut-originated species of *Bifidobacterium* and *Lactobacillus* were demonstrated to have inhibitory effects on *P. larvae* [42]. These bacteria produce organic acids that increase the acidity of the digestive tract and inhibit the growth of pathogenic microorganisms [43–45]. In addition, the *Bifidobacterium* and *Lactobacillus* bacteria enhance the honey bee immune system by upregulating antimicrobial peptides in honey bees [46]. However, in this study, only *Lactobacillus* sp. showed inhibitory properties against *P. larvae* growth on agar plates (Table S1; Figure S2). The *Bifidobacterium* strains showed no inhibition against *P. larvae* (strain ATCC9545) ERIC genotype I [47] used in this study. The result was consistent with that of Forsgren et al. [42] who showed that the strains of *Bifidobacterium* had an antimicrobial effect against only the ERIC genotype III and IV of *P. larvae* and no inhibition against ERIC I.

The LAB isolated from the honey bee gut has been demonstrated to be helpful for the inhibition of *P. larvae* [48]. The LAB species with an inhibitory effect against *P. larvae* were previously isolated from the gut of *A. mellifera* L. and identified as strains of 11 species in the genus *Lactobacillus* (*L. kunkeei*, *L. plantatarum, L. apinorum, L. mellis, L. kimbladii, L. kullabergensis*, *L. mellifer*, *L. apis*, *L. helsingborgensis*, *L. brevis*, and *L. melliventris*) [25, 29, 30, 42, 48, 49], and strains of two species in genus *Bifidobacterium (B. asteroides* and *B. coryneforme*) [42, 48]. Five of the six *Lactobacillus* species with inhibitory effects against *P. larvae*, which were identified in this study, were similar to those reported in previous studies. Notably, another species was newly determined, *L. panisapium*. This species was isolated from both larva and adult bees. Therefore, the species could be safe to apply in both living stages of honey bees.

The differences in the structure of the digestive tract and food sources between the adult and larval stages in honey bees could result in the difference in gut microbiome between the two stages [21, 50, 51]. Therefore, it could be harmful when the larvae receive a large amount of LAB isolated from adult bees. It was demonstrated that the brood size was reduced when the LAB mixture was supplementally administered to the colonies for *P. larvae* treatment [52]. Consequently, LAB originating from larvae may be the optimal probiotic candidate for safe application in the colony. The *L. panisapium* PKH2_L3 strain isolated from larvae in this study with antimicrobial effect against *P. larvae* and no harm to larvae could be selected for further evaluation as a potential probiotic candidate.

The combination of all isolated strains of different LAB was expected to increase the diversity of metabolites and antimicrobial peptides that help inhibit *P. larvae* [42, 48]. However, the influence of each LAB strain and the mixture on the healthy larvae were not previously evaluated. Feeding larvae with the combination of six strains in different species isolated in this study also showed high efficiency of *P. larvae* inhibition compared to feeding larvae with individual strains. However, the survival rate of larvae fed with the combination of six LAB was lower than that of the group that was singly fed with only *L. apis* HSY8_B25, *L. panisapium* PKH2_L3, *L. mellis* OMG2_B33, *L. melliventris* HSY3_B5, or *L. kimbladii* AHS3_B36 (Fig. 4). In addition, administration of the six LAB strains to the healthy larvae (no *P. larvae* infection) demonstrated that some species help enhance the survival rate of the larvae, such as *L. apis* HSY8_B25, *L. mellis* OMG2_B33, and *L. melliventris* HSY3_B5. However, *L. kimbladii* AHS3_B36 is harmful to the larvae (Fig. 3). Therefore, the combination of all LAB strains could result in an increase in larval mortality, and safety screening is necessary to select the potential LAB strain for AFB treatment.

Adhesion ability is an essential property of the desirable probiotic bacteria to demonstrate effective antimicrobial activity [53]. The bacteria with high adhesion ability can prevent elimination by peristalsis and develop in the host intestinal tract to protect against the colonization of other harmful microorganisms [25, 28, 54]. The potentiality of the bacteria to adhere to the intestinal tract of honey bees can be tested by its adhesion to hydrocarbon (BATH), and the hydrophobicity and auto-aggregation ability are helpful in understanding the adherence ability of the probiotic bacteria [55, 56]. Among the six strains selected in this study as probiotic candidates, two species (*L. kullabergensis* OMG2_B25 and *L. mellis* OMG2_B33) demonstrated low adhesion to toluene and xylene, and the other four species had moderate to high adhesion ability in comparison with other probiotic species [54, 57]. The auto-aggregation of the six species was higher than those of other probiotic candidates for honey bees analyzed in previous studies [25]. Therefore, the four strains (*L. apis* HSY8_B25, *L. panisapium* PKH2_L3, *L. melliventris* HSY3_B5, and *L. kimbladii* AHS3_B36) with high adhesion ability could be selected for further analysis.

## Conclusions

Isolation of gut bacteria from honey bees was conducted to find bacteria with antimicrobial effects against *P. larvae*; 20 of the 67 isolated strains belonging to only the *Lactobacillus* genus showed inhibitory properties to *P. larvae* on agar plates. Six representative strains from six different species (*L. apis* HSY8_B25, *L. panisapium* PKH2_L3, *L. melliventris* HSY3_B5, *L. kimbladii* AHS3_B36, *L. kullabergensis* OMG2_B25, and *L. mellis* OMG2_B33) were selected for evaluation of the probiotic properties, of which three species (*L. apis* HSY8_B25, *L. panisapium* PKH2_L3, and *L. melliventris* HSY3_B5) passed the tests for probiotic properties, including safety to larvae, inhibition of *P. larvae* growth in infected larvae, and adhesion ability. These strains could be potential candidates for probiotics to control AFB disease.

## Methods

### Isolation of the gut microbiome from honey bees

Live honey bees (*Apis mellifera* L.) in the combs were collected from 18 different apiaries in South Korea and carried to the laboratory. Subsequently, they were washed twice using autoclaved distilled water. Overall, 90 gut samples (larvae [*n* = 36] and adult bees [*n* = 54]) collected from 18 different apiaries were isolated (Supplementary Figure S1). The three guts of adult bees or two guts of larvae collected from each apiary were placed into a tissue grinding tube with steel beads (SNC, Hanam, Korea). Next, 500 µL phosphate-buffered saline (PBS) solution was added, and the samples were vortexed for 10 s. The homogenate was briefly centrifuged at 200 ×g for 30 s; subsequently, 100 µL of the supernatant was spread on different agar plates as follows: brain heart infusion (BHI), and the De Man, Rogosa and Sharpe (MRS) [58]. The plates were incubated at 37 ℃ in an anaerobic condition until white round colonies were seen (24–72 h). The anaerobic jar system Anoxomat AN2CTS (MART Microbiology B.V., 9207 JB Drachten, Netherlands) was used to provide an anaerobic condition. Finally, the colonies were selected from agar plates and transferred to a broth medium for cultivation in the same condition for further analysis.

### Species identification of isolated microbiome

The colonies of bacteria BHI or MRS agar were singly selected and inoculated in broth medium for 24 h at 37 ℃ in anaerobic conditions. Stock bacteria were made from the cultivated bacteria by adding glycerol to the final 20% and stored at -80℃, and the remaining solution of each cultivation was used for species identification. Bacteria from the medium were collected by centrifuging at 13,000 ×g for 5 min and discarding the supernatant. Deoxyribonucleic acid (DNA) was extracted using the FastDNA Spin Kit for Soil (MP Biochemicals GmbH, Eschwege, Germany) following the manufacturer’s instructions. The 16S rRNA gene of bacteria was amplified and sequenced using primer pair 518F: 5’-CCA GCA GCC GCG GTA ATA CG-3’/800R: 5’-TAC CAG GGT ATC TAA TCC-3’ (Macrogen, Inc., Seoul, South Korea). The sequences were analyzed by comparing them with the National Center for Biotechnology Information (NCBI) database using the Basic Local Alignment Search Tool (BLAST-NCBI). Notably, sequences on NCBI with the highest similarity to that of isolated strains were selected for alignment using Clustal X version 2.0 [59]. A maximum likelihood phylogenetic tree was constructed using the Kimura 2-parameter model [60], gamma distribution, and bootstrapping 500 times with the software MEGA version 7 [61].

### Screening of antimicrobial activity

The inhibition assay was conducted according to a previous method [62] with some modifications. The vegetative form of *P. larvae* ATCC 9545 strain, enterobacterial repetitive intergenic consensus (ERIC) genotype I, was cultivated at 35 ℃ for 48 h in a BHI medium. The concentration adjustment of OD_600_ = 0.7 was made, and the cultivated medium of *P. larvae* was spread on a BHI agar plate using a sterile cotton swab, and three replicate plates were used. The isolated strains of LAB were cultured on MRS agar at 37 ℃ for 3 days. Afterwards, a single colony was selected and cultured in MRS broth for 18 h at 37 ℃ in an anaerobic condition. The LAB in cultured MRS broth were collected by centrifugation at 13,000 ×g for 5 min, and the supernatant was discarded. The pellet was washed twice by suspending it in PBS solution and centrifuging under the same condition. Finally, the pellet was suspended by PBS and adjusted to OD_600_ = 0.7; subsequently, 10 µL of the suspended bacterial solution was dispensed onto a sterile filter paper (6 mm, Whatman, USA) and placed on a BHI agar plate, where the *P. larvae* were spread. The inhibition zone was observed after 48 h incubation at 37 ℃, microaerobic condition, 5% CO_2_.

### Safety examination of the isolated LAB on honey bee larvae

The representative strain of each species with the largest inhibition zone was selected for the safety examination of in vitro rearing larvae. LAB strains were cultured on MRS agar at 37 ℃ in an anaerobic condition for 3 days; a single colony was selected and cultured in MRS broth for 18 h at 37 ℃ in an anaerobic condition. The cultivated bacteria were collected by centrifugation at 13,000 ×g for 5 min, and the bacterial pellet was suspended by feeding diet to a concentration of 1 × 10^4^ cells/µL [63]. The feeding diet was prepared with 6% glucose, 6% fructose, 1% yeast extract, and 50% royal jelly [64]. Larvae (1 to 2 instar) of *A. mellifera* L. were transferred to a 6-well plate with 10 larvae/well. Each LAB strain was evaluated with larvae in three wells (*n* = 30). Feeding solution of 200 µL containing LAB (1 × 10^4^ cells/µL) was supplied to the larvae in one dose on day 1; subsequently, the larvae were fed daily with feeding solution without LAB until day 7. The control groups were fed with feeding solution without LAB. Finally, the number of dead larvae was recorded daily. Notably, dead larvae were identified by lack of body elasticity or color change to brown.

### Inhibition of ***Paenibacillus larvae*** in artificially infected larvae

The antibacterial effect of the isolated LAB was evaluated in an in vivo larval model. Honey bee larvae (*A. mellifera* L.) 1 to 2 instar were transferred to a 48-well plate, one larva/well. Larvae in each group, including three plates (*n* = 144), were used for each LAB strain. One group was fed with a mixture of six selected LAB strains, another group was infected with only *P. larvae* with no LAB administration, and the control group was fed with only feeding solution without *P. larvae* and LAB. The feeding diet containing LAB was prepared as described above. In preparing *P. larvae* spores for infection, colonies of *P. larvae* were suspended in BHI broth, then spread on Columbia sheep blood agar (BD, Franklin Lakes, NJ, USA) and incubated for 10 days at 37 ℃. A medium containing spores was collected, and the spores were suspended in a feeding solution with 1,000 spores/µL. The larvae were supplied by a feeding diet containing LAB in one dose (50 µL/larva) on day 2, and a feeding diet containing *P. larvae* spores (50 µL/larva) was supplied to each larva on day 3. Then, 50 µL of feeding diet was supplied daily to each larva until day 5. The larvae were inspected daily, and the number of dead larvae in each group was recorded. At the end of the period, three living larvae of each group were randomly selected for real-time PCR detection of *P. larvae* using primer pairs AFB-F: AAA TCA TCA TGC CCC TTA TG/ AFB-R: CGA TTA CTA GCA ATT CCG ACT, and the probe: FAM-CGT ACT ACA ATG GCC GGT ACA ACG–BHQ-1 [65]. The digestive tract of the larvae was separated after washing five times with autoclaved distilled water and used for DNA extraction employing the FastDNA Spin Kit for Soil (MP Biochemicals GmbH, Eschwege, Germany) following the manufacturer’s instructions. Finally, 50 µL of DNA solution was acquired from each group. The PCR reaction mix (20 µL) was composed of 1 µL (10 pmol) of each primer, 1 µL (5 pmol) of the probe, 2 µL of the extracted DNA solution, 5 µL of ddH_2_O, and 10 µL of Iq™ supermix as PCR premix (Bio-Rad, Hercules, CA, USA). PCR was performed at 95 ℃ for 5 min, followed by 40 PCR cycles for 10 s at 95 ℃ and 30 s at 60 ℃. DNA copy of *P. larvae* was calculated based on the cycle threshold (Ct) of *P. larvae* detection from each group using a standard linear regression. Standard curves (Supplementary Figure S3) representing the relationship between Ct value and initial DNA copy were established from the amplification using *P. larvae* recombinant DNA 10^8^-10^1^ copies (10-fold dilution).

### Hydrophobicity and auto-aggregation

Adhesion ability is an essential property of desired probiotics by which the bacteria can adhere to intestinal epithelial cells [54]. The ability of cells to adhere to the intestine can be affected by the composition and structure of the cell surface, and the hydrophobicity of the cell surface is known to be a major factor [66]. Therefore, the auto-aggregation and hydrophobicity ability of the LAB were measured. Auto-aggregation capacity was evaluated according to a previously reported method [54]. The LAB strains were cultured at 37 ℃ for 18 h under anaerobic conditions. The LAB bacteria were collected by centrifugation at 8,000 ×g, at 4 ℃, for 10 min, and were washed twice in PBS. The bacteria were suspended in PBS and adjusted to a concentration of OD_580_ = 0.5. The bacteria suspensions were incubated at 37 ℃, and the OD_580_ was measured at 0, 1, 5, 18, and 24 h, respectively. The auto-aggregation percentage was calculated using the formula: Auto-aggregation % = [1 – (OD_final_/OD_0_)] × 100, in which OD_0_ was measured at the time 0 h, and OD final was measured at 1, 5, 18, and 24 h [67], respectively. On the other hand, the hydrophobicity ability of LAB was evaluated by its ability to adhere to the hydrocarbons xylene and toluene [25]. Preparation of LAB was done as described above. After measuring the OD_580_ = 0.5 ± 0.05, xylene or toluene was added to each washed bacterial strain in a ratio of 1:1 (v/v). The mixtures were vortexed for 2 min and incubated for 60 min at room temperature, and the aqueous phase was removed for measurement of OD_580_. Hydrophobicity was calculated using the formula:

Hydrophobicity % = [1 – OD_final_/OD_0_] × 100, where OD_final_ and OD_0_ represent the absorbance values after 60 min incubation and before adding xylene or toluene, respectively. The level of hydrophobicity was classified as low (0–35%), moderate (36–70%), or high (71–100%) [57].

### Statistical analysis

Survival rates of larvae were analyzed using the Kaplan–Meier method [68, 69]. To compare the results among the six strains in the auto-aggregation test, the survival rate of larvae in the safety examination, and the *P. larvae* DNA levels in the infected groups that received different strains of LAB, one-way analysis of variance (ANOVA) was performed. The PAST version 4.03 software was used [70]. The statistical level of significance was set at *p* < 0.05.

**Tables**.

## Electronic supplementary material

Below is the link to the electronic supplementary material.

Supplementary Material 1

## Data Availability

All data generated or analyzed during this study are included in this article and its supplementary information files.

## Declarations

### Supplementary Information

Additional file 1: Table S1. Antimicrobial activity of isolated lactic acid bacteria against *P. larvae*.

Additional file 2: Figure S1. Extraction of the digestive tract from adult bees and larvae; Figure S2. Antimicrobial activity of the isolated bacteria against *P. larvae*; Figure S3. Standard curves of *P. larvae* DNA amplification.

## Abbreviations

AFB: American foulbrood
LAB: lactic acid bacteria
BATH: Bacterial Adherence to Hydrocarbons
AA: auto-aggregation
CT: cycle threshold
PCR: polymerase chain reaction
OD: optical density
ANOVA: analysis of variance
DNA: Deoxyribonucleic acid
ERIC: Enterobacterial repetitive intergenic consensus
