# Supplementary material for: Probiotic candidates for controlling Paenibacillus larvae, a causative agent of American foulbrood disease in honey bee
Source: BMC Microbiol. 2023 May 24;23:150. doi: 10.1186/s12866-023-02902-0 (PMC10207761; doi:10.1186/s12866-023-02902-0)
Supplement: Supplementary file 1 — Supplementary Material 1 [file 12866_2023_2902_MOESM1_ESM.pdf]

Table S1. Antimicrobial activity of isolated lactic acid bacteria against *P. larvae*

No.	Isolate name	Species name	Phylum	Inhibition zone (mm)	Antimicrobial activity	Host
1	KGS1_B26	*Gilliamella apicola*	*Proteobacteria*	0.0 ± 0.0	-	Adult bee
2	HSY8_B22	*Enterobacter* sp.		0.0 ± 0.0	-	Adult bee
3	LKS5_B24	*Bifidobacterium asteroides*	*Actinobacteria*	0.0 ± 0.0	-	Adult bee
4	LKS5_B26	*Bifidobacterium asteroides*		0.0 ± 0.0	-	Adult bee
5	HSY3_B8	*Bifidobacterium asteroides*		0.0 ± 0.0	-	Adult bee
6	OMG2_B28-1	*Bifidobacterium asteroides*		0.0 ± 0.0	-	Adult bee
7	LKS5_B31	*Bifidobacterium* sp.		0.0 ± 0.0	-	Adult bee
8	LKS5_B32	*Bifidobacterium asteroides*		0.0 ± 0.0	-	Adult bee
9	LKS5_B38	*Bifidobacterium* sp.		0.0 ± 0.0	-	Adult bee
10	LKS5_B41	*Bifidobacterium* sp.		0.0 ± 0.0	-	Adult bee
11	PKH1_B15	*Bifidobacterium* sp.		0.0 ± 0.0	-	Adult bee
12	OMG2_B39	*Bifidobacterium* sp.		0.0 ± 0.0	-	Adult bee
13	OMG2_B40	*Bifidobacterium* sp.		0.0 ± 0.0	-	Adult bee
14	OMG2_B44	*Bifidobacterium* sp.		0.0 ± 0.0	-	Adult bee
15	AHS3_B25	*Bifidobacterium* sp.		0.0 ± 0.0	-	Adult bee
16	MSJ1_B1	*Bifidobacterium* sp.		0.0 ± 0.0	-	Adult bee
17	MSJ1_B16	*Bifidobacterium* sp.		0.0 ± 0.0	-	Adult bee
18	MSJ1_B19	*Bifidobacterium* sp.		0.0 ± 0.0	-	Adult bee
19	MSJ1_B15	*Bifidobacterium indicum*		0.0 ± 0.0	-	Adult bee
20	MSJ1_B17	*Bifidobacterium indicum*		0.0 ± 0.0	-	Adult bee
21	OMG2_B38	*Bifidobacterium* sp.		0.0 ± 0.0	-	Adult bee
22	OMG2_B43	*Bifidobacterium* sp.		0.0 ± 0.0	-	Adult bee
23	AHS3_B16	*Bifidobacterium* sp.		0.0 ± 0.0	-	Adult bee
24	HSY8_B50	*Bifidobacterium* sp.		0.0 ± 0.0	-	Adult bee
25	AHS3_B13_1	*Bifidobacterium* sp.		0.0 ± 0.0	-	Adult bee
26	HSY8_B20	*Bifidobacterium* sp.		0.0 ± 0.0	-	Adult bee
27	PKH1_B13	*Lactobacillus* sp.	*Firmicutes*	0.0 ± 0.0	-	Adult bee
28	OMG2_B25	*Lactobacillus kullabergensis*		12.7±2.3	+	Adult bee
29	PKH1_B16	*Lactobacillus* sp.		0.0 ± 0.0	-	Adult bee
30	PKH1_B13-3	*Lactobacillus* sp.		0.0 ± 0.0	-	Adult bee
31	OMG2_B19	*Lactobacillus* sp.		9.3±1.2	+	Adult bee
32	PKH2_B3	*Lactobacillus* sp.		0.0 ± 0.0	-	Adult bee
33	PKH2_B4	*Lactobacillus* sp.		0.0 ± 0.0	-	Adult bee
34	PKH2_B5	*Lactobacillus* sp.		0.0 ± 0.0	-	Adult bee
35	HSY8_B25	*Lactobacillus apis*		18.0±2.0	+	Adult bee
36	PKH2_B6	*Lactobacillus* sp.		0.0 ± 0.0	-	Adult bee
37	KJA1_B19	*Lactobacillus* sp.		0.0 ± 0.0	-	Adult bee
38	HSY8_B18	*Lactobacillus apis*		10.7±1.2	+	Adult bee
39	HSY8_B33	*Lactobacillus apis*		0.0 ± 0.0	-	Adult bee
40	PKH2_L3	*Lactobacillus panisapium*		11.3±1.2	+	Larvae
41	HSY8_B27	*Lactobacillus apis*		0.0 ± 0.0	-	Adult bee
42	KJA1_B18	*Lactobacillus* sp.		6.0±5.3	+	Adult bee
43	MSJ1_B8	*Lactobacillus panisapium*		0.0 ± 0.0	-	Adult bee
44	MSJ1_B11	*Lactobacillus panisapium*		10.7±1.2	+	Adult bee
45	MSJ1_B13-2	*Lactobacillus panisapium*		8.7±1.2	+	Adult bee
46	MSJ1_B23	*Lactobacillus panisapium*		0.0 ± 0.0	-	Adult bee
47	MSJ1_B25-2	*Lactobacillus panisapium*		10.0±0	+	Adult bee
48	MSJ1_B27	*Lactobacillus panisapium*		9.3±1.2	+	Adult bee
49	MSJ1_B29	*Lactobacillus panisapium*		0.0 ± 0.0	-	Adult bee
50	MSJ1_B31	*Lactobacillus panisapium*		0.0 ± 0.0	-	Adult bee
51	MSJ1_B30-1	*Lactobacillus melliventris*		12.0±2.0	+	Adult bee
52	AHS3_B7	*Lactobacillus* sp.		15.3±1.2	+	Adult bee
53	PKH2_B8	*Lactobacillus* sp.		0.0 ± 0.0	-	Adult bee
54	PKH2_B18	*Lactobacillus mellis*		0.0 ± 0.0	-	Adult bee
55	HSY8_B17	*Lactobacillus mellis*		12.0±0	+	Adult bee
56	OMG2_B32	*Lactobacillus* sp.		0.0 ± 0.0	-	Adult bee
57	HSY3_B4	*Lactobacillus melliventris*		0.0 ± 0.0	-	Adult bee
58	PKH2_L5_3	*Lactobacillus* sp.		0.0 ± 0.0	-	Larvae
59	PKH2_B2	*Lactobacillus* sp.		0.0 ± 0.0	-	Adult bee
60	OMG2_B33	*Lactobacillus mellis*		12.7±1.2	+	Adult bee
61	AHS3_B13-2	*Lactobacillus* sp.		20.0±0	+	Adult bee
62	AHS3_B33	*Lactobacillus* sp.		12.0±0	+	Adult bee
63	KJA1_B17	*Lactobacillus* sp.		13.3±2.3	+	Adult bee
64	KJA1_B10	*Lactobacillus* sp.		18.7±6.4	+	Adult bee
65	HSY3_B5	*Lactobacillus melliventris*		20.0±0	+	Adult bee
66	AHS3_B36	*Lactobacillus kimbladii*		20.0±0	+	Adult bee
67	KGS1_B21	*Lactobacillus* sp.		0.0 ± 0.0	-	Adult bee

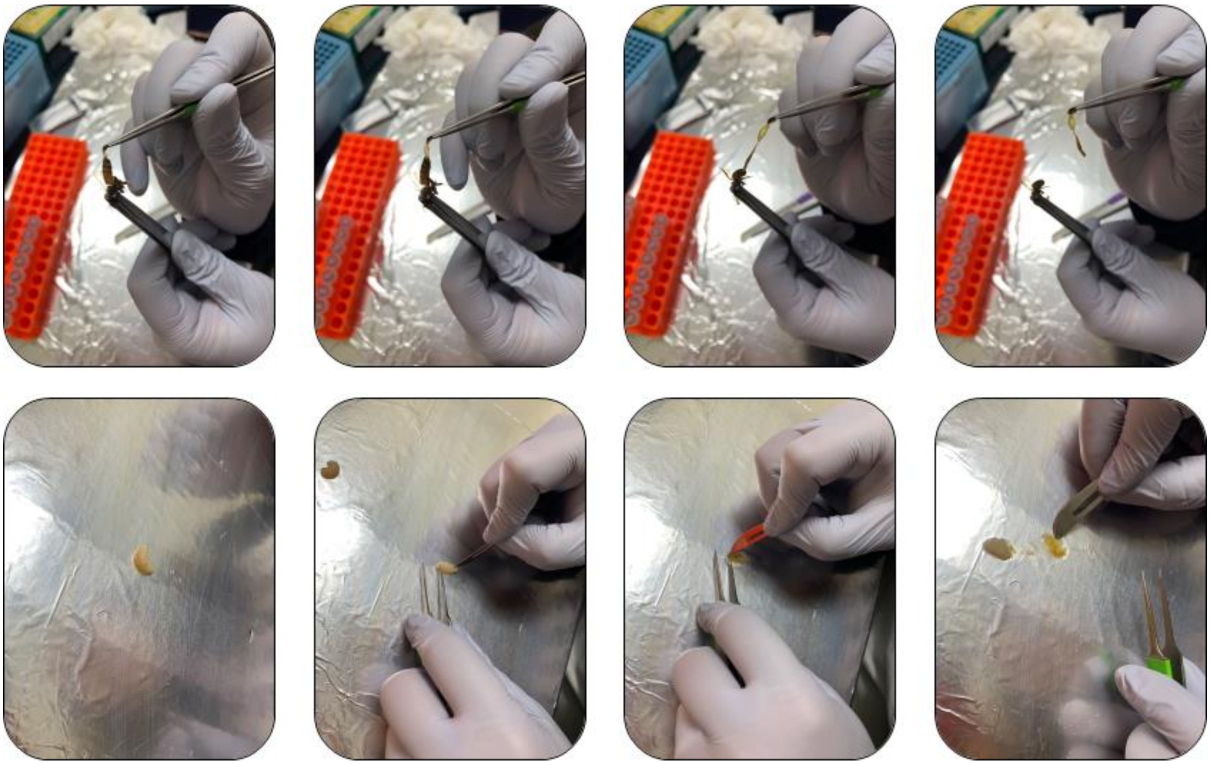

Figure S1. Extraction of the digestive tract from adult bees and larvae. The guts of adult bees were pulled out using sterilized tweezers. Larvae were dissected on an autoclaved aluminum foil using scalpels and tweezers. The guts were then transferred to tissue grinding tubes with steel beads and PBS solution. The samples were handled on a clean bench.

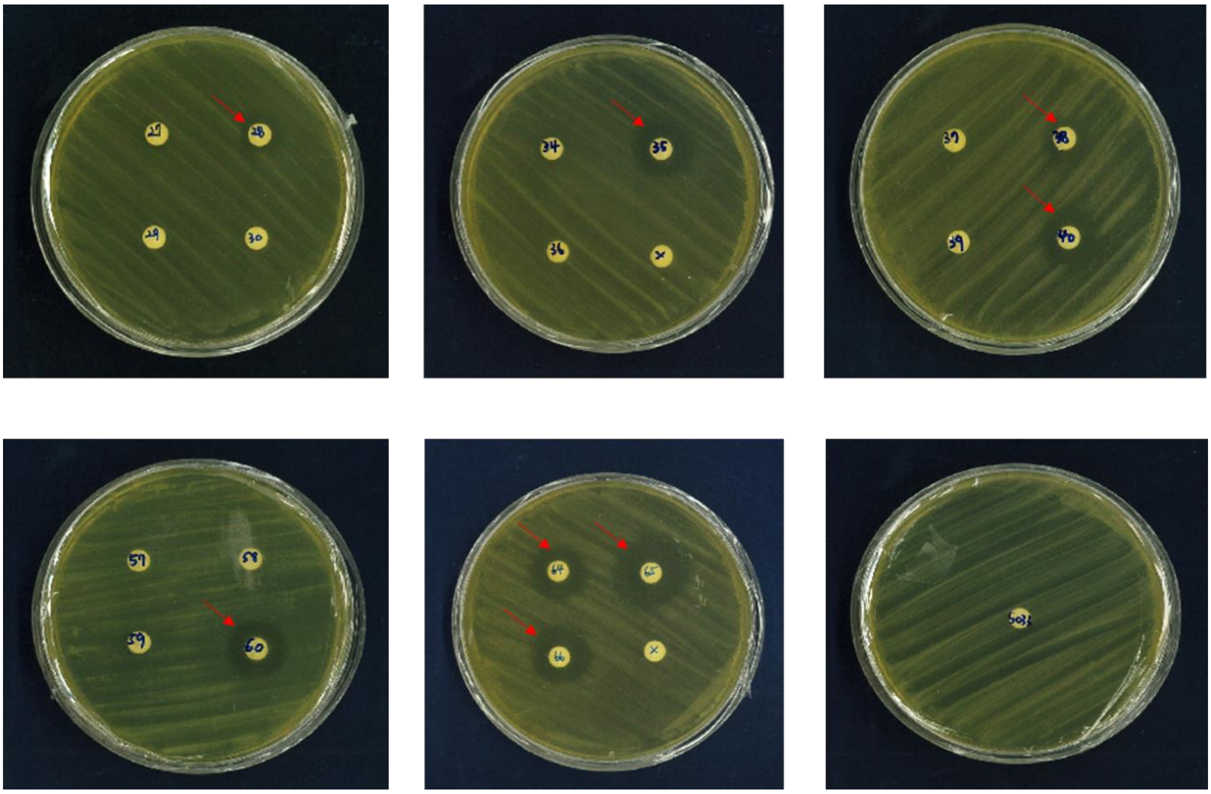

Figure S2. Antimicrobial activity of the isolated bacteria against *P. larvae*. The antimicrobial effect of isolated bacteria was identified by inhibiting the growth of *P*. *larvae* with an inhibition zone observed surrounding the plotted position of isolated strains on the MRS agar plate, indicated by red arrows. The number of isolates is shown; 5033 was the control strain *Lactobacillus rhamnosus* KCTC 5033.

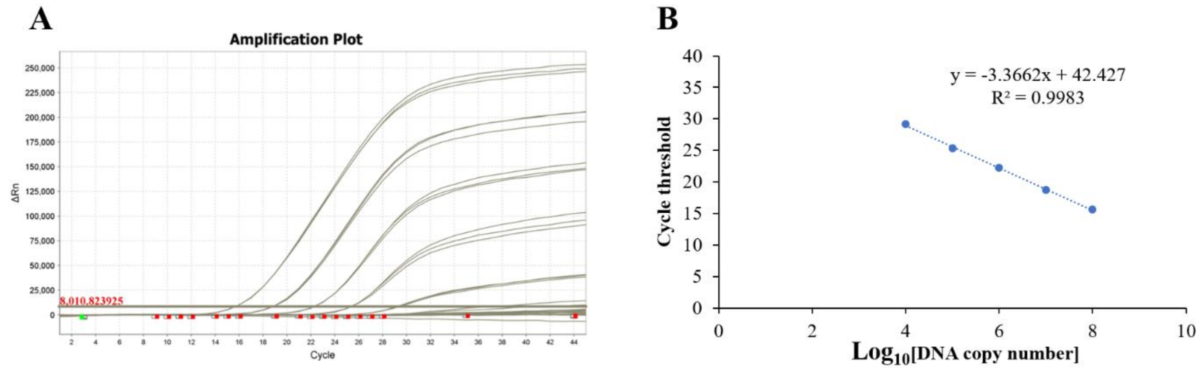

Figure S3. Standard curves of *P. larvae* DNA amplification. Ten-fold serial dilution of *P. larvae* recombinant DNA, from 10^{10} to 10^{1} copies, was used for triplicate PCRs (**A**). Correlation between the initial number of DNA copies ($\log_{10}$-transformed values) and the cycle threshold of amplification is shown (**B**).
